# An Updated Overview on Nanonutraceuticals: Focus on Nanoprebiotics and Nanoprobiotics

**DOI:** 10.3390/ijms21072285

**Published:** 2020-03-26

**Authors:** Alessandra Durazzo, Amirhossein Nazhand, Massimo Lucarini, Atanas G. Atanasov, Eliana B. Souto, Ettore Novellino, Raffaele Capasso, Antonello Santini

**Affiliations:** 1 CREA—Research Centre for Food and Nutrition; Via Ardeatina 546, 00178 Rome, Italy; alessandra.durazzo@crea.gov.it (A.D.); massimo.lucarini@crea.gov.it (M.L.); 2 Biotechnology Department, Sari University of Agricultural Sciences and Natural Resources, 9th km of Farah Abad Road, Mazandaran, 48181 68984 Sari, Iran; nazhand.ah@gmail.com; 3 Institute of Neurobiology, Bulgarian Academy of Sciences, 23 Acad. G. Bonchev str., 1113 Sofia, Bulgaria; atanas.atanasov@gmail.com; 4 Institute of Genetics and Animal Breeding, Polish Academy of Sciences, Jastrzębiec, 05-552 Magdalenka, Poland; 5 Department of Pharmacognosy, University of Vienna, Althanstraße 14, 1090 Vienna, Austria; 6 Ludwig Boltzmann Institute for Digital Health and Patient Safety, Medical University of Vienna, Spitalgasse 23, 1090 Vienna, Austria; 7 Department of Pharmaceutical Technology, Faculty of Pharmacy, University of Coimbra, Pólo das Ciências da Saúde, 3000-548 Coimbra, Portugal; ebsouto@ebsouto.pt; 8 CEB-Centre of Biological Engineering, University of Minho, Campus de Gualtar, 4710-057 Braga, Portugal; 9 Department of Pharmacy, University of Napoli Federico II, Via D. Montesano 49, 80131 Napoli, Italy; ettore.novellino@unina.it; 10 Department of Agricultural Sciences, University of Napoli Federico II, Via Università 100, 80055 Portici (Napoli), Italy

**Keywords:** nutraceuticals, nanotechnologies, nanonutraceuticals, prebiotics, probiotics, synbiotics

## Abstract

Over the last few years, the application of nanotechnology to nutraceuticals has been rapidly growing due to its ability to enhance the bioavailability of the loaded active ingredients, resulting in improved therapeutic/nutraceutical outcomes. The focus of this work is nanoprebiotics and nanoprobiotics, terms which stand for the loading of a set of compounds (e.g., prebiotics, probiotics, and synbiotics) in nanoparticles that work as absorption enhancers in the gastrointestinal tract. In this manuscript, the main features of prebiotics and probiotics are highlighted, together with the discussion of emerging applications of nanotechnologies in their formulation. Current research strategies are also discussed, in particular the promising use of nanofibers for the delivery of probiotics. Synbiotic-based nanoparticles represent an innovative trend within this area of interest. As only few experimental studies on nanoprebiotics and nanoprobiotics are available in the scientific literature, research on this prominent field is needed, covering effectiveness, bioavailability, and safety aspects.

## 1. Nanonutraceuticals

### 1.1. Nutraceuticals

Beside the emerging need for natural origin alternatives to pharmaceuticals, the interest is focusing more and more on possible applications of food derived products that can be used as tools to prevent (and in some cases also cure) or delay the onset of a health issue [1,2,3]. Nutraceuticals, are a novel toolbox not completely explored so far for its full potential in medicine [4,5,6,7]. Nutraceuticals, a portmanteau of the words ‘nutrition’ and ‘pharmaceutical’ [2], have been defined as “the phytocomplex if they derive from a food of vegetal origin, and as the pool of the secondary metabolites if they derive from a food of animal origin, concentrated and administered in the more suitable pharmaceutical form” [8]. Examples of substances that have nutritional and nutraceutical interest are antioxidants, vitamins, polyunsaturated fatty acids, dietary fibres, prebiotics, and probiotics [9]. Nutraceuticals reside nowadays in a gray area between pharmaceuticals and food; their safety and efficacy in health conditions and safety must be substantiated by clinical data; moreover, there is lack of a shared regulatory system for them [7,10].

### 1.2. From Nanopharmaceuticals to Nanonutraceuticals

#### 1.2.1. Characteristics of Nanoparticles and General Classification

Within the different definitions of nanomaterials, these can be described as the products of nanotechnology, characterized by at least one dimension within the size range below 100 nanometers [11,12,13]. Due to their remarkable properties and versatility, nanomaterials are being exploited in different fields, e.g., agriculture, health, electronics, cosmetics [14,15,16,17,18], representing a great challenge, in particular, in food science and technology, environment, and human health [19]. The progress in pharmaceutical nanotechnology has led to a new class of products, the so-called nanopharmaceuticals [20,21], defined as pharmaceutical drug molecules formulated in nanomaterials. Different types of nanoformulations are being exploited for the treatment of neurodegenerative diseases, cancer, infectious diseases, and others [22,23,24,25,26]. Besides, nanomaterials are also succeeding in offering new advanced tools for imaging and diagnosis [27] which, combined with therapy, have been proposed as nanotheranostics. These formulations are also being tailored for personalized medicine.

Nanoparticles can be produced from natural (e.g., proteins, polysaccharides, lipids) and from synthetic (e.g., polymers) sources. Ideally, materials should be biocompatible, biodegradable, and biotolerable, namely the way by which designed materials are tolerated by the body, and of generally recognized as safe (GRAS) status, in order to be used in pharmaceutical and nutraceutical products. Among the available options, and if the nanoparticles are intended for oral administration (as happens with nanonutraceuticals), lipid nanoparticles are of special interest [28,29,30,31]. Lipids are known for their role as absorption enhancers in the gut, which contribute to improving the oral bioavailability of several drugs and biomolecules. Besides this, the loading of poorly soluble drugs into lipid nanoparticles overcome the limitations encountered in their formulation into final products. Lipid nanoparticles can be produced from well-known lipids existing both in the human body and in foodstuff (e.g., fatty acids, triglycerides, phospholipids, waxes, cholesterol) thereby enhancing their biodegradability, and biocompatibility profiles [32].

Among polysaccharides, chitosan [33,34,35,36,37] and alginate [33,38,39], have been frequently used in the production of nanoparticles for oral delivery. Being a mucoadhesive polysaccharide, chitosan is able to increase cellular permeability and improves the bioavailability of orally administered drugs and proteins. Moreover, the molecule itself exhibits antimicrobial properties, and has a low toxicity. The molecule has chemical functional groups that can be modified for site specific targeting. Alginate is also a versatile mucoadhesive natural polymer with very low toxicity in vivo. Alginate nanoparticles have a hydrophilic character with improved loading capacity for hydrophilic drugs, being able to modify their release profile. Alginate nanoparticles are reported as adjuvants in vaccinations and can be produced conjugated with dextran to modify the release profile of proteins and other macromolecules intended for oral administration [40].

Nanopharmaceuticals and nanonutraceuticals are obtained, respectively, when a pharmaceutical or a nutraceutical is formulated in nanoparticles. The rationale for their development is mainly addressed to improve the physicochemical properties (e.g., solubility) and pharmacokinetic parameters (t_max_, C_max_, area under the plasma drug concentration–time curve (AUC)), with the ultimate aim to reduce the dose required to observe the therapeutic/nutraceutical outcome and thus the possible risk of toxicity [41,42,43]. Parameters, such as efficiency, quality, and safety should therefore be considered. Nevertheless, regulatory issues related to nanopharmaceuticals still need further developments [44].

#### 1.2.2. Emerging Area of Applications

Nanopharmaceuticals and the great change of the pharmaceutical industry have a great impact also on nutraceuticals. The recent work of Agarwal et al. [45] gives the patented and approval scenario of nanopharmaceuticals with regards to biomedical application, manufacturing procedure, and safety aspects.

Wu et al. [46] highlighted how nanotherapeutics and nanopharmaceuticals could lead to a more precise individual diagnosis, improve targeted therapies, reduce side effects, and enhance therapeutic monitoring. The same review also underlines that the field of nanomedicine is at its early stage and that further efforts to translate their potential into clinical trials and medical practice are still needed.

A growing number of studies are addressed towards the application of nanotechnologies to nutraceuticals [47,48,49,50] in order to obtain improved bioavailability, delivery, and effect. This leads to the development of an emerging area of innovative products: the nanonutraceuticals [51,52,53].

Nanotechnology can be used to improve absorption, bioavailability, stability, and controlled release of nutrients and nutraceuticals, thereby increasing health benefits; some examples of potential advantages of applications of nanotechnology on the nutraceuticals are (i) efficient encapsulation; (ii) smart delivery and release from a nanoformulation. For example, research on encapsulation of nutraceuticals into biodegradable, environmentally friendly nanocarriers, is ongoing to increase their absorption and their therapeutic potential.

The nanonutraceutical formulations represent a valuable and promising strategy to maintain nutraceutical health beneficial properties at a nano level, to guarantee safety and efficacy, when used in managing health conditions, particularly for patients who are not eligible for a conventional pharmacological therapy. Follow-up studies, as reported by recent works [54,55,56,57], and communication strategies [58], are needed for both the nanopharmaceuticals and nanonutraceuticals [59,60], in view of expanding the area of interest to different health conditions. For instance, Aditya et al. [61] describe the current status of the various delivery systems that are used for the delivery of hydrophilic bioactive compounds and discuss future prospects to be explored for the delivery of hydrophilic bioactive compoundse.g., niosomes, bilosomes, cubosomes.

## 2. Focus on Nanotechnologies Applied to Prebiotics, Probiotics, and Synbiotics

Focus of this perspective is the application of nanotechnologies to food supplements containing prebiotics, probiotics, and synbiotics. This section consists of (i) shot on prebiotics, probiotics, and synbiotics; (ii) definition and delineation of nano-prebiotics, nano-probiotics, and nano-synbiotics.

### 2.1. An Overview on Prebiotics, Probiotics, and Synbiotics

#### 2.1.1. Prebiotics

**Figure 1:**
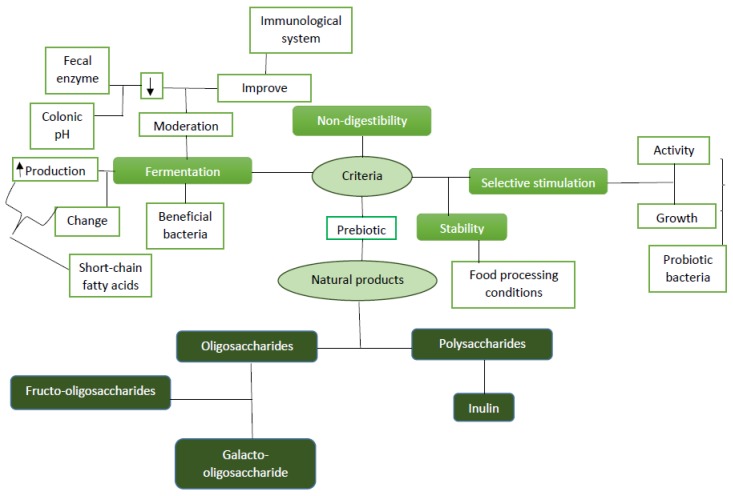
Overview of prebiotics.

Prebiotics [62,63,64,65,66] are a special form of dietary fiber with health benefits, which invoke alterations in the host microbial ecosystem, not only in the gut, via their selective administration by live host microbes [67]. Food ingredients like prebiotics are classified on the basis of some principles, such as resistance to digestion in upper alimentary tract, selective stimulation of probiotic growth, beneficial health effects in the host, stability in different conditions of food/feed processing, and fermentation process through intestinal microbiota. They are found in various sources, including some non-digestible oligosaccharides, non-digestible carbohydrates, yacon, unrefined wheat, unrefined barley, soybeans, raw oats, breast milk, and inulin sources (e.g., chicory roots and Jerusalem artichoke) [68]. Some compounds found in prebiotics are soya-oligosaccharide, xylo-oligosaccharide, pyrodextrins, gluco-oligosaccharide, lactulose, malto-oligosaccharide, galactans (galacto-oligosaccharide (GOS)), oligofructose, isomalto-oligosaccharide (IOS), fructans (FOS and inulin), mannan-oligosaccharide (MOS), lactitol, and non-starch polysaccharides (NSP). Figure 1 gives an overview of prebiotics.

**Figure 2:**
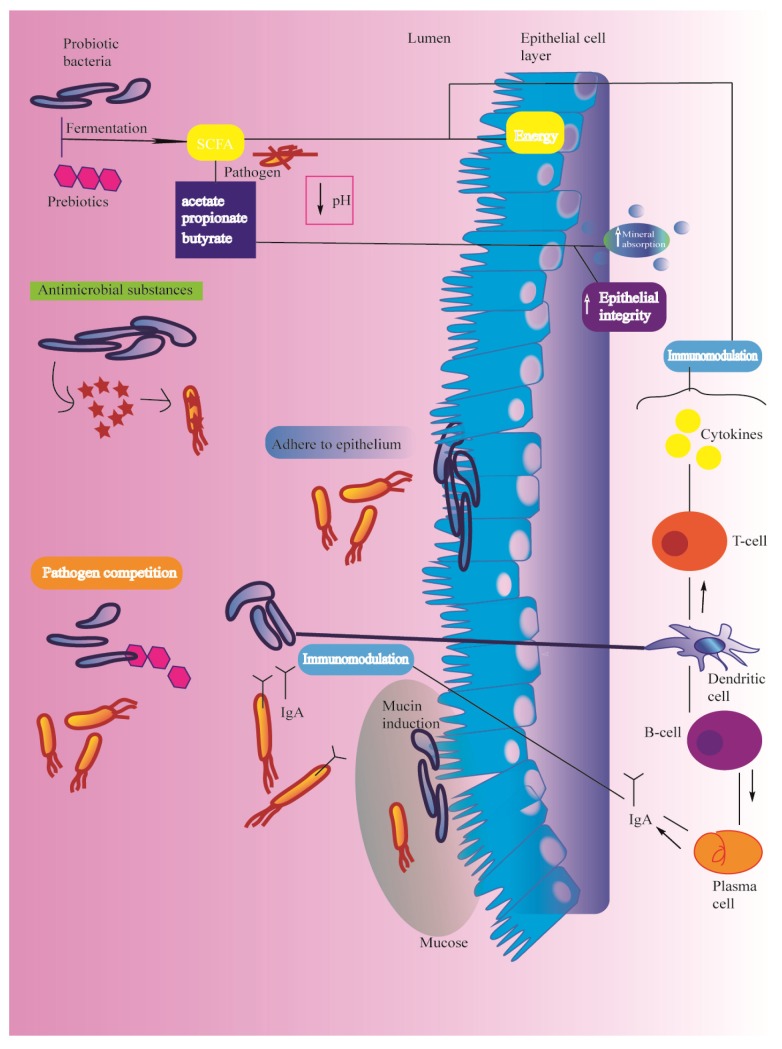
Overview of mechanism of action of pre and probiotics.

Several mechanisms are involved in the bioactivity of prebiotics and probiotics [69,70], as described in Figure 2.

The metabolic products of such microorganisms can drop the gastrointestinal (GI) pH by carbohydrate fermentation via *Bifidobacteria* and *Lactobacillus* thereby influencing mineral uptake, growth, and spread of gut microbiota, epithelial integrity, and hormonal regulation. They also are able to enhance the absorption of trace elements and especially of iron and act on the regulation of body immune function. The prebiotics can use the short-chain fatty acids (SCFAs) as an energy source.

#### 2.1.2. Probiotics

**Figure 3:**
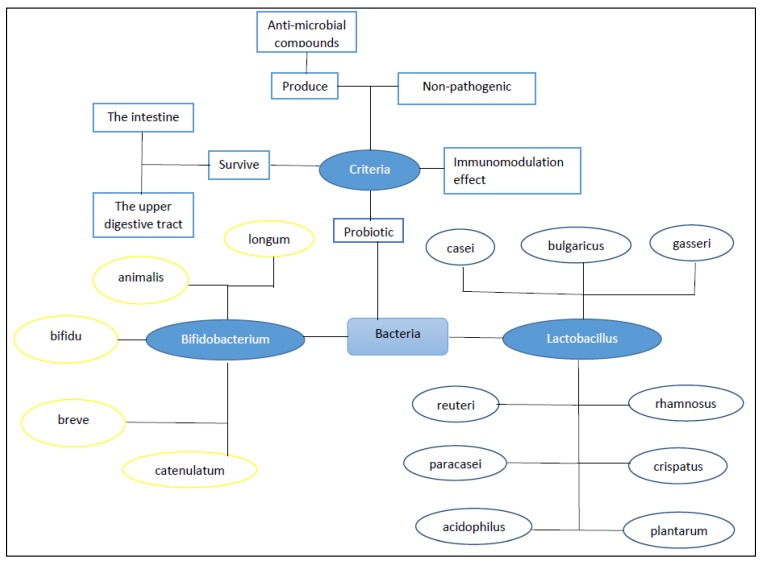
An overview of probiotics.

The FAO (Food and Agriculture Organization) and WHO (World Health Organization) have defined probiotics as non-pathogenic living microorganisms that ensure host health if used properly in foods or as dietary supplements [71,72]. Probiotics come from different sources, such as various natural environments, human gut microbiota, and foods. The main properties of probiotics like the ability to survive through the gastrointestinal tract, the resistance against bile and gastric acidity, and the stimulation of the activity of bile salt hydrolase, promote health benefits to the host [68,73,74,75,76,77,78,79,80,81]. The count of probiotic bacteria (colony-forming units (CFU)/g) in probiotic-containing products differ among the countries; for example, 10^7^ CFU/g in the USA and 10^9^ CFU/g in Canada. The effective dose generally contains >10^6^–10^8^ CFU/g or >10^8^–10^10^ CFU/d of live probiotic bacteria [82,83]. Most probiotics are found in Gram-positive bacteria, including *Streptococcus*, *Bacillus*, *Lactobacillus*, *Enterococcus*, and *Pediococcus*. The probiotics can also include fungal and yeast species such as *Saccharomyces cerevisiae* and *Kluyveromyces*. Only some microorganisms such as *Lactobacillus* spp., *Bifidobacterium* spp., and *Lactococcus* are known as generally recognized as safe (GRAS) despite the existence of diverse microorganisms which can act as probiotics with health benefits [84,85,86]. Figure 3 gives an overview of probiotics.

The reported key mechanisms of action of probiotics [87] have been mentioned as follows (see Figure 2): enhancement of epithelial barrier, modulation of insulin-sensitive tissues, synthesis of antimicrobial substances, multi-pathogen competition, and induction of mucin secretion. The probiotics are able to adhere to epithelium, resulting in microbial elimination. They also modulate the immune function via the stimulation of signaling pathways to upregulate anti-inflammatory cytokines and growth factors, to differentiate T-regulatory cells (Tregs), and to interact with the gut-brain axis (GBA) by endocrine regulation and neurologic functions.

#### 2.1.3. Synbiotics

The synbiotic agents are a combination of prebiotics and probiotics with beneficial effects on host through the enhancement of activity and survival of beneficial microorganisms in the gastrointestinal tract, so that they can selectively provoke the growth and stimulate the metabolism of one or more health-promoting bacteria, thereby enhancing the host welfare [88,89,90,91,92,93,94,95,96,97]. The most important issue in the design of synbiotics resides in the prebiotic and probiotic selection criteria and requirements, which should be clearly described.

#### 2.1.4. Health Promoting Effect of Prebiotics, Probiotics, and Synbiotics

The International Scientific Association for Probiotics and Prebiotics (ISAPP) introduced a wide range of products containing the probiotics with health promoting effects, including non-edible products (e.g., vaginal preparations), baby formulas (e.g., first milk), drugs, therapeutic supplements (e.g., for enteral nutrition), and foods (e.g., fermented milk with reportedly health beneficial effects) [98].

Some of the reported beneficial effects of probiotics in human health include anticancer [99,100,101,102,103,104,105,106,107,108,109,110,111], anti-allergic [112,113], anti-diabetic [114,115,116], anti-obesity [117,118,119,120], anti-pathogenic [121,122], immunomodulatory [123], and anti-inflammatory [124,125,126,127] activities [128], as reported in Table 1. In an in vitro study, Sequential Window Acquisition of All Theoretical Mass Spectra (SWATH-MS) as a quantitative analysis technique was applied to evaluate the proteomic profile of colon cancer cells in *Lactobacillus kefiri SGL 13*, and the results indicated antiproliferative and pro-apoptotic activities for this strain on human colon adenocarcinoma cell line HT29 [99]. In another study, the airway hyper reactivity was suppressed in ovalbumin-sensitized samples by *Lactobacillus* spp. (such as *Lactobacillus* and *Pediococcus*) via a reduction in the level of Th2 cytokines, OVA-specific IgE and IgG1 as well as an increase in the level of IgG2a [112]. *Lactobacillus fermentum* cell-free supernatant (LCFS) caused cancer cell death in 3D HCT-116 conditions through the induction of apoptosis in the colon cancer cell line and the antiproliferative activity by the inhibition of NF-κB signaling [129]. The use of lactoferrin and *Bifidobacterium longum BB536* managed the enteropathy caused by diclofenac in rat samples by modulating the proinflammatory pathway of TLR-2/-4/NF-kB [130]. Othman et al. [131], studied the effect of inactivated *Bifidobacterium longum* intake on obese diabetes affected mice. They reported a significant decrease of body weight gain, adipose tissue mass and blood glucose levels, as well as a significant reduction in blood glucose after a 5 weeks treatment. The treatment also resulted in reduced levels of cholesterol and triglycerides [131].

The administration of three strains of *Bifidobacteria* in the adult rats improved neuronal plasticity and cognitive behavior [132].

Prebiotics have been reported to have different activities; for example, generation of bacteriocins, maintenance of gut health [133], possibility to be used as food additive and starter culture, clearance of cholesterol [134,135], potentiation of immune defense [136], inhibition of constipation and risk of obesity [137,138], inhibition of colitis [139], protection of colon and other organs against cancer [140,141,142], reduction of cardiovascular disease risk factors, antioxidant activity [143,144], over-bioavailability [145]. According to scientific published data, the administration of oligofructose-enriched inulin (OEI) promotes malondialdehyde content, lipid profile, glycemic indices, and antioxidant level in female patients suffering from type II diabetes [146]. The supplementation of inulin in shaken cultures was found to increase the growth rate of *L. plantarum ST16* [147]. Based on the findings from Ramos et al. [148], the administration of fructooligosaccharides (FOS) was tolerated and decreased the total and free p-cresyl sulfate (PCS) in the serum samples of patients with non-diabetic chronic kidney disease (NDD-CKD).

The therapeutic potential of synbiotics has been comprehensively discussed in a recent review published by Flesch et al. [149]. According to their findings, the patients with irritable bowel syndrome (IBS) when receiving *B. longum BB536* and *L. rhamnosus HN001* plus vitamin B6 showed restoration of intestinal permeability and gut microbiota, as well as amelioration of the disease symptoms [150]. In the research of Mohan et al., the synbiotic AMF^TM^ 15^+^ manuka honey yogurt showed antibacterial properties, followed by increasing probiotic bacteria and producing lactic and propionic acids [151]. A study reported gut health enhancement following the administration of seaweed-based synbiotic of *Gracilaria coronopifolia* which caused the reduction of inflammation, the generation of reactive oxygen species (ROS), and diminution of the oxidative stress-induced cell damage [152]. According to Sarwar et al., the textural properties, such as adhesiveness, cohesiveness, and hardness, were enhanced following the co-administration of inulin and *Saccharomyces boulardii* [153]. In Table 1 an updated overview of in vitro and in vivo studies on prebiotic, probiotic, and synbiotic products is given.

### 2.2. Nano-Prebiotics, Nano-Probiotics, and Nano-Synbiotics

Recently, emerging applications of nanotechnologies in prebiotics and probiotics have been developed and carried out as reported in Table 2 [154,155,156,157,158,159,160,161,162,163,164,165,166,167].

Caneus et al. [168] remarked how nanomedicine, together with the known practices of prebiotics, probiotics, and synbiotics, represents a valuable approach in creating an optimal environment within the gastrointestinal tract.

Exploring the nanonization strategies of probiotics and the utility of nanoprobiotics in the delivery of encapsulated bacteria is being carried out. For encapsulation of probiotic have been used mainly nanoparticles i.e., with of selenium and gold particles of a size in the range 10–1000 nm; nanolayers, consisting of at least three layers of a charged polyelectrolyte, a polymeric layer, and a functionalized polysaccharide or polyether; nanoemulsions consisting of a liquid phase dispersion in another liquid phase with droplet size less 200 nm; nanobeads (nanosized bacteria-enabled autonomous delivery system) and emerging product of nanofibers [169]. The best technique for probiotics encapsulation was mainly chosen for protecting the cells against an adverse environment in the gastrointestinal tract, in order to allow their release in a viable and metabolically active state in the intestine [170].

Kazmierczak et al. [171] describe an innovative engineering approach to load such nanoparticles onto a biological “mailman” (a novel, nontoxic, therapeutic strain of *Salmonella typhimurium* engineered to preferentially and precisely seek out, penetrate, and hinder prostate cancer cells as biological delivery system) that will deliver the therapeutics to a target site. Another example of probiotic bacteria encapsulated with nanoparticles was given by Hu et al. [172] that showed how coating live bacterial cells with synthetic nanoparticles represents a promising strategy to engineer efficient and versatile DNA vaccines. Feher et al. [173] have reported the use of nano-sized particles of probiotics for preventing and treating neuroinflammation.

Probiotics are indeed receiving special interest as an alternative to the classical antibiotics to overcome bacterial resistance. As prebiotics enhance the activity of probiotics, Kim et al. [162] proposed the development of a prebiotic formulation composed of *Pediococcus acidilactidi* loaded in phthalyl dextran nanoparticles by conjugating phthalic anhydride with dextran [162]. The authors evaluated the cellular effects of the produced nanomaterial and checked the antimicrobial properties of the probiotics. The loading of *P. acidilactidi* into phthalyl dextran nanoparticles was found to enhance the production of antimicrobial peptides by probiotics by a self-defense mechanism, with improved antimicrobial effect against Gram (+) and Gram (−) micro-organisms compared to the probiotics alone. The same authors previously reported that prebiotic phthalyl inulin nanoparticles could also enhance the antimicrobial activities of *P. acidilactici* [174].

Hong et al. also reported the enhanced antimicrobial activity of phthalyl pullulan nanoparticles treated with *L. plantarum* against *Escherichia coli* K99 and *Listeria monocytogenes* [164]. The nanoparticles were internalized into the *L. plantarum* by an energy-dependent and galactose transporter-dependent mechanism and a higher amount of plantaricin, a natural antibacterial peptide, was secreted from the developed nanoprobiotic than from probiotic alone.

The use of spores from probiotics have been recently proposed as a delivery system for chemotherapeutic drugs. Song et al. [175] produced deoxycholic acid-modified spores to be loaded with doxorubicin and sorafenib as an approach for autonomous production of nanoparticles in the gastrointestinal tract. Such approach envisions drug protection upon oral administration to improve bioavailability. Besides, the release is based on the disintegrated hydrophobic protein and the hydrophilic deoxycholic acid with enhanced uptake by the epithelial cells via the bile acid pathway, increasing basolateral drug release.

The anticancer activity of silver/*Lactobacillus rhamnosus GG* nanoparticles was described by Aziz et al. [155]. Using the MTT assay, the authors demonstrated that the viability of HT-29 cell lines has been significantly reduced when applying the highest tested nanoparticle concentration, leading to apoptosis. The method of synthesizing silver/*Lactobacillus rhamnosus GG* nanoparticles was also found to be cost-effective, offering a viable nanoprobiotic approach for biomedical applications.

It is worth mentioning the work of Fung et al. [176] where, by investigating the agrowaste-based nanofibers as a probiotic encapsulant, has proposed the use of nanofibers for the nanoencapsulation of *L. acidophilus* using 8% poly(vinyl alcohol) to produce nanofibers by electrospinning technology. The authors suggested how thermal behavior of nanofibers suggested possible thermal protection of probiotics in heat-processed foods. Nagy et al. [177] by investigating the suitability of electrospinning for biodrugs delivery to produce vaginal drug delivery systems, concluded how nanofibers can provide long term stability for huge amounts of living bacteria if they are kept at (or below) 7 °C. The recent work of Zupancic et al. [178], who studied the incorporation of a range of safe lactic acid bacteria into poly(ethylene oxide)-based nanofibers, evidenced that all of the lactic acid bacteria were viable after incorporation into nanofibers, with 0–3 log CFU/mg loss in viability, depending on the species. Moreover, the authors reported that viability can be correlated with the hydrophobicity and to the extreme length of lactic acid bacteria, whereas a horizontal or vertical electrospinning set-up did not have any role. Development of nanofibers via electrospinning has a great potential and use in pharmaceutical and food industry for their properties i.e., sterile nature, biocompatibility, adhesiveness, efficiency, and as vehicle for controlled and sustained release in drug delivery [179,180,181,182]. Electrospinning and electrospraying represent innovative technologies for the delivery of nutraceuticals [183].

An example of nanolayers coated probiotics has been given by Franz et al. [184] who developed layer-by-layer nano self-assembly coating of *Allochromatium vinosum* with different polyelectrolyte combinations and investigated substrate uptake in bacteria: surface charge neither affected sulfide uptake nor the contact formation between the cells and solid sulfur, whereas increasing layers slowed or inhibited the uptake of sulfide and elemental sulfur.

The recent work of Ebrahimnejad et al. [185] described the use of chitosan for nanoencapsulation of *L. acidophilus* as probiotic bacteria, by concluding how nanoencapsulation of probiotic bacteria represents a promising strategy in enhancing the viability and survival of them against gastro-intestinal environmental conditions.

Ranjan et al. [186] reported physicochemical characterization and potential prebiotic effect of whey protein isolate/inulin nano complex.

Atia et al. [167] developed an encapsulated oral-symbiotic supplement by studying the effect of adding inulin in alginate beads and observed their ability to protect three probiotic strains, namely, *P. acidilactici*, *L. reuteri*, and *L. salivarius*. The antimicrobial and probiotic properties of bacterial strains were found not to be affected by the encapsulation.

Krithika and Preetha [166] have developed a protein-based inulin incorporated symbiotic nanoemulsion for enhanced stability of probiotic; whey protein concentrate/inulin nano complex can be recommended as a delivery system for various probiotics in food products.

Salmerón et al. [187] reported the development fermented beverages with synbiotic properties, and the incorporation of nanoparticles with unique and specific bioactivity, to improve organoleptic characteristics, absorption, and delivery of nutrients and bioactive compounds which has opened a new horizon in this segment of food created to improve human health and well-being.

Formulation of protein-based inulin incorporated synbiotic nanoemulsion for enhanced stability of probiotic are currently studied extensively.

It is worth mentioning the work of Rezaee et al. [188] that investigated the antimicrobial activity of Ag and TiO_2_ nano-particles on three species of *Lactobacillus* i.e., *L. casei ATCC 39392*, *L. plantarum ATCC 8014*, and *L. fermentum ATCC 9338* in the presence and absence of raffinose, lactulose, and inulin, respectively. The results indicated that silver nanoparticles decreased 85%, 85%, and 71% of *L. casei*, *L. plantarum*, and *L. fermentum*, respectively, after 48 h and decreased percentages of *L. casei*, *L. plantarum,* and *L. fermentum* that were 16%, 64%, and 4% in the presence of the prebiotics. Nano TiO_2_ particles decreased 59%, 85%, and 61% of *L. casei*, *L. plantarum,* and *L. fermentum*, respectively, after 48 h, and decreased percentages of *L. casei*, *L. plantarum,* and *L. fermentum* which were 16%, 2%, and 4% in the presence of these prebiotics.

The treatment of gastrointestinal disorders (e.g., diarrhea) using nanoprobiotics is also a relatively unexplored field. Khan et al. [189] aimed at quantifying the concentration of nanomaterials commercialized in chocolates and evaluated their effect on a commercial probiotic formulation (containing *Bacillus coagulans*, *Enterococcus faecalis*, and *Enterococcus faecium*) usually used to treat diarrhea in children [189]. The known probiotic activities, such as acid production, biofilm formation, growth, and antibiotic resistance were observed from isolated bacteria, while the isolated titanium oxide nanoparticles from chocolates were shown to inhibit the growth and activity of the probiotic formulation in a concentration range of 125–500 µg/mL in vitro [189]. The outcomes of this study concluded that TiO_2_ in chocolate discourages the survival of probiotic bacteria in the gastrointestinal tract.

To trace target probiotics in situ and in real-time, Liu et al. [190] developed an in vivo probing strategy using persistent luminescence nanophosphors surface-modified by plasmid-like DNA as optical labelling and background-free fluorescence bioimaging as signal readout. The surface modification with DNA molecules was shown to promote the nanoparticles penetration into the bacteria and facilitated in vivo bioimaging. Such an approach opens new research perspectives in terms of food safety making use of nanotechnologies.

## 3. Conclusions

Only a few experimental studies are present in literature on nanoprebiotics and nanoprobiotics, while studies on this prominent issue are needed, covering effectiveness and safety aspects as it has been developed for pharmaceuticals. The potential of nanotechnologies in the food area is an emerging challenge as well as the nanonutraceuticals, which are an emerging field of study in the nutraceuticals area. Safety and regulatory aspects should be considered to depict the potentiality of nanoprobiotics and nanoprebiotics. Nanoformulation should be accompanied with regulatory requirements to ensure efficacy, safety, and authorization procedures. As a general guideline, the European Authority for Food Safety (EFSA) [191] has developed an approach for assessing the potential risks arising from the applications of nanoscience and nanotechnologies in the food and feed chain. Regarding prebiotics and probiotics, McClements and Xiao [192] developed a summary of the possible applications of inorganic and organic nanoparticles in foods, a description of the nanoparticle characteristics, and discussed the importance of the food matrix and gastrointestinal tract effects on nanoparticle properties as well as potential possible toxicity mechanisms of different food-grade nanoparticles. The same authors concluded, however, that many of these nanoparticles are unlikely to have adverse-side effects on human health in line with previously reported data [193]. Nonetheless, in order to assess the effective use of food-grade nanoparticles, further studies are expected to exploit and assess safety, improved bioavailability, and efficacy.

## Figures and Tables

**Table 1:** An updated overview of in vitro and in vivo studies on prebiotic, probiotic, and synbiotic products.

Type	Microorganisms/Prebiotics	Activity	Study	References
Probiotic	Bacillus and Enterobacter	Anticancer and antioxidant effect	The intracellular cell-free supernatants (CFS) from *Bacillus licheniformis* KT921419 and the ethyl acetate extracts could control the growth of HT-29, a colon cancer cell line	[100]
	*L. plantarum C70*	Anticancer effect	*L. plantarum* C70 by releasing the exopolysaccharide caused 73.1% and 88.1% cytotoxic properties against the breast and colon cancers, respectively	[101]
	*Kluyveromyces marxianus* and *Pichia kudriavzevii*	Anticancer effect	According to analysis of Annexin V/PI and DAPI, an apoptotic induction was observed due to exopolysaccharides released by probiotic yeasts of *Kluyveromyces marxianus* and *Pichia kudriavzevii*	[102]
	*Lactobacilli cocktail*	Anticancer effect	HT-29, a human colorectal carcinoma cell line was controlled by *Lactobacilli cocktail* via the modulation of the Notch and Wnt/β-catenin signaling pathways	[104]
	*L. rhamnosus*	Anticancer effect	The bioconversion of cranberry proanthocyanidins to *Lactobacillus rhamnosus* could result in the IC_50_ values of 20.1 and 47.8 μg/mL	[105]
	*Bifidobacterium infantis, L. acidophilus, Enterococcus faecalis, Bacillus cereus*	Anti-inflammatory effect	A mixture of aerobic probiotics improved the functions of various intestinal barriers and the restoration of lucrative intestinal microbiota in the mouse model of DSS-induced chronic colitis, meaning anti-inflammatory properties	[127]
	*Saccharomyces boulardii* CNCM I-745	Anti-inflammatory effect	The inflammatory response was modulated in mucositis caused by 5-FU (fluorouracil) via the probiotic *Saccharomyces boulardii* CNCM I-745 through the control of TLR 2 and 4 as well as the reduction of pro-inflammatory and NF-κB cytokines	[103]
	*L. casei* IMAU60214	Immunomodulatory effect	The use of *L. casei* IMAU60214 killed by heat increased the activity of M1-like pro-inflammatory phenotype through the TLR2 signaling pathway	[123]
	*L. plantarum*	Antimicrobial effect	*L. plantarum* ZLP001 impeded the ETEC adhesion and linked with IPEC-J2 cells via the competition and exclusion	[122]
	Lactobacillus	Anti-diabetic effect	The lactobacillus strain alleviated the levels of blood sugar and HbA1c in diabetic rats	[115]
	*L. plantarum* LMT1-48	Anti-obesity effect	The body weight and abdominal fat content were decreased in mouse models fed a modified diet through the administration of *L. plantarum* LMT1-48 at a density of 10^6^ CFU/mL	[117]
	*Hafnia alvei*	Anti-obesity effect	Fat mass, food intake, and body weights were reduced in the mouse model of obesity and hyperphagia	[118]
	*Eurotium cristatum*	Anti-obesity effect	The administration of *Eurotium cristatum* showed anti-obesity activity in mice fed a high-fat diet (HFD) through the modulation of gut microbiota	[119]
	*L. plantarum* strain TCI378	Anti-obesity	The expression of glucose transporter type 4 (GLUT-4) and adipocyte-specific genes perilipin 1 was suppressed by metabolism derivatives from *L. plantarum* strain TCI378	[120]
Prebiotic	Galacto-oligosaccharides and phycocyanin	Anticancer effect	The prebiotics co-administered by phycocyanin arrested the cell cycle at the G0/G1 phase, resulting in inhibited growth of HCT116 cells	[141]
	Chondroitin Sulfate Disaccharide	Anticancer effect	The growth of HT-29, human colon cancer cell line, was controlled by Chondroitin sulfate (CS)-Keel disaccharide (CSD) generated by chondroitin AC lyase, estimating at 80% antiproliferative activity	[140]
	Short-chain fatty acids	Antiproliferative effects	The administration of short-chain fatty acids (SCFAs) prevented the expression of genes involved in human colorectal cancer cells	[142]
	Blueberry anthocyanins	Antioxidant effect	The density and composition of intestinal microbiota in human models were increased by consumption of high purity blueberry anthocyanins through the increase in the modulatory and prebiotic activities	[143]
	Oligosaccharides	Antioxidant effect	The water-soluble oligosaccharide of EMOS-1a showed 1420% proliferation level	[144]
	*Lycium barbarum* polysaccharide	Immunomodulation effect	The administration of polysaccharides derived from *Lycium barbarum* in mice showed immunomodulatory effects, and enhanced density of beneficial bacteria and gut microbiota	[136]
Synbiotic	Djulis (*Chenopodium formosanum*) with *L. acidophilus*	Anticancer effect	The co-administration of Djulis (*Chenopodium formosanum* Koidz.) and *Lactobacillus acidophilus* inhibited the growth of rat colon cancer cells through the promotion of apoptosis, proliferation, and inflammation	[80]
	*L. casei, acidophilus, rhamnosus, bulgaricus, Bifidobacterium breve, longum* and *Streptococcus thermophilus* with fructo-oligosaccharides.	Anticancer and antioxidant effect	Ten weeks of low-calorie diet program along with synbiotic supplementation enhanced the activity of superoxide dismutase (SOD) and reduced the serum level of malondialdehyde (MDA) in obese patients suffering from breast cancer-related lymphedema	[89]
	*Weissella cibaria* FB069 with xylooligosaccharides	Anticancer effect	The use of synbiotic-fermented soymilk (containing xylooligosaccharides and *Weissella cibaria* FB069) inhibited the proliferation of HCT116 and Caco-2, colorectal cancer cell lines, through the reduction in the transcription of MD2/TLR4/MyD88/NF-κB	[90]
	*Auricularia auricula aqueous* with *L. acidophilus* La-5 and *Bifidobacterium bifidum* Bb-12	Antioxidant effect	The aqueous extract of *Auricularia auricula* in the presence of *L. acidophilus* La-5 and *Bifidobacterium bifidum* Bb-12 significantly elevated the level of phenolic compounds and the activity of antioxidant properties up to 1057.6 mg of Gallic acid/kg and 115.30 of mg BHT eq/kg following 28-day storage	[91]
	*L. bulgaricus PXN 39, L. casei subsp. casei PXN 37, Bifidobacterium breve PXN 25, L. rhamnosus PXN 54, B. infantis PXN 27 Lactobacillus acidophilus PXN 35, Streptococcus thermophilus PXN 66* with fructo-oligosaccharides	Immunomodulation effect	The use of multispecies symbiotic showed immunoregulatory effects on the expression levels of CD4 and IgA in mice exposed to lipopolysaccharide (LPS)	[92]
	*L. plantarum* with inulin	Neuropsychological effect	Concomitant administration of inulin and *L. plantarum* in diabetic rats improved CREB/BDNF/TrkB signaling pathway, serotonin secretion, brain parameters, intestinal microbial composition, and oxidative stress, thus leading to improved memory and learning disorders	[93]
	β-glucan, *Bacillus coagulans*, and inulin, lactic acid	Anti-diabetic effect	Eight weeks of taking daily synbiotic plus lactic acid improved the levels of GSH-Px, SOD and HbA1c in patients with type II diabetes	[94]
	Corn starch, maltodextrin, inulin, fructooligosaccharides, potassium chloride, magnesium sulfate, mangan sulfate with *L. casei W56, acidophilus W22, paracasei W20, salivarius W24, plantarum W62, Lactococcus lactis W19, Bifidobacterium lactis W51* and W52, and *Bifidobacterium bifidum W23*	Improve symptoms of diarrhea-predominant irritable bowel syndrome	Irritable bowel syndrome (IBS) symptoms were improved by synbiotic treatment through an increase in fecal acetate and butyrate, colonic CD4+ T cells, mucosal microbial diversity as well as a decrease in surrogate of intestinal barrier function and fecal zonulin	[95]
	Grape pomace extract with lactobacilli	Anti- inflammatory effect	The co-administration of lactobacilli and prebiotic grape pomace caused a downregulation of inflammatory genes, proteins, signaling molecules through the symbiotic effects	[96]
	*L. acidophilus, L. rhamnosus, B. longum* and *Bifidobacterium bifidum, Saccharomyces boulardii* with fructo-oligosaccharides	Hepatoprotective effects	The administration of synbiotic soy yogurt controlled hypercholesterolemia in mice liver by reducing the levels of low-density lipoprotein cholesterol, triacylglycerols, blood cholesterol, and lipid peroxidation.	[97]

**Table 2:** Emerging applications of nanotechnologies on nanoprobiotics, nanoprebiotics, and nano synbiotics.

Type	Activity	Study	References
Probiotic	Antimicrobial effect	The polylysine-induced poly glutamic acid (PG) films caused protection of probiotics against food-borne pathogens	[154]
	Anticancer effect	The high levels of synthesized silver/*Lactobacillus rhamnosus* GG nanoparticles (Ag-LNPs) led to a decline in the rate of HT-29 live cells	[155]
	Anticancer and antimicrobial effect	The fabrication of copper oxide nanoparticles (CuO-NPs) using *L. casei* could control the proliferation of HT-29, a human colon carcinoma cell line, and human gastric carcinoma cell line, as well as could eliminate *Pseudomonas aeruginosa* and *Staphylococcus aureus*	[156]
	Anticancer and antioxidant effect	The *L. casei* capped-SeNPS suppressed the cytotoxicity caused by Diquat and oxidative damage, impeded the cell damage and apoptosis induced by H_2_O_2_, and induced the apoptosis mediated by the HepG2 cell line	[157]
	Anticancer and antioxidant effect	The findings from the administration of *L. casei* 393-SeNPs were the induction of HepG2 cell line apoptosis, the elevation of oxidative damage caused by Diquat in IECs, and the reduction in gut barrier dysfunction caused by ETEC K88 via the antioxidant functions, the regulation of inflammation, the establishment of gut epithelial barrier integrity, and the balance of gut microflora	[158]
	Anticancer effect	Dead nano-scale *L. plantarum* could impede the proliferation of a colorectal cancer cell line through an increase in the expression level of IgA, an induction of cancer cell cycle arrest and apoptosis, and a suppression of inflammatory response	[159]
	Anticancer and antioxidant effect	The synthesis gold nanoparticles (AuNps) having antioxidant activity and low cytotoxicity using *L. kimchicus* DCY51T strain exhibited the activity of a protective protein capping layer	[160]
Prebiotic	Improve drug delivery	High molecular weight (HMW) inulin nanoparticles were fabricated to achieve drug delivery system, whose concentration of <200 μg/mL had no toxicity for peripheral blood mononuclear cells (PBMCs)	[161]
	Antimicrobial effect	The probiotics were internalized by phthalyl dextran nanoparticles (PDNs) to construct pediocin, aiming at the alteration of gut microbiome composition, the suppression of pathogenic intestinal infections, and the elevation of beneficial bacteria species	[162]
	Antimicrobial effect	The higher pediocin generation following the administration of PIN-internalized probiotics with 0.171 polydispersity index (PDI) with a size of about 203 nm showed the maximum antimicrobial properties	[163]
Synbiotic	Antimicrobial effect	The activity of Listeria monocytogenes and *Escherichia coli K99* was inhibited by *L. plantarum* exposed to phthalylpullulan nanoparticle (PPN) due to production of antimicrobial peptides via intracellular stimulation	[164]
	The photo protective effect	A cream containing *L. rhamnosus* plus Selenium nanoparticles could heal the side effects induced by sunburn and showed sun protection factor (SPF) of 29.77 in Wistar rat model	[165]
	Improve delivery system	A new formulation of nano-emulsion containing *E. faecium* plus inulin could increase probiotic bacterial viability and stability	[166]
	Improve tolerance of probiotic bacteria	Beads reinforced by inulin (5% *w*/*v*) had the highest effect on bacterial protection against bile salts	[167]
